# Toll-Like Receptor 4 Triggering Promotes Cytosolic Routing of DC-SIGN-Targeted Antigens for Presentation on MHC Class I

**DOI:** 10.3389/fimmu.2018.01231

**Published:** 2018-06-14

**Authors:** Sophie K. Horrevorts, Sanne Duinkerken, Karien Bloem, Pablo Secades, Hakan Kalay, René J. Musters, Sandra J. van Vliet, Juan J. García-Vallejo, Yvette van Kooyk

**Affiliations:** ^1^Department of Molecular Cell Biology and Immunology, Cancer Center Amsterdam, VU University Medical Center, Amsterdam, Netherlands; ^2^Centre for Specialized Nutrition, Danone Research, Wageningen, Netherlands; ^3^Division of Cell Biology, Dutch Cancer Institute, Amsterdam, Netherlands; ^4^Department of Physiology, VU University Medical Center, Amsterdam, Netherlands

**Keywords:** dendritic cells, DC-SIGN, cross-presentation, imaging flow cytometry, toll-like receptor, MHC-I, T cell, proteasome

## Abstract

DC-SIGN is an antigen uptake receptor expressed on dendritic cells (DCs) with specificity for glycans present on a broad variety of pathogens and is capable of directing its cargo to MHC-I and MHC-II pathways for the induction of CD8^+^ and CD4^+^ T cell responses, respectively. Therefore, DC-SIGN is a very promising target for the delivery of antigen for anti-cancer vaccination. Although the endocytic route leading to MHC-II presentation is characterized to a large extent, the mechanisms controlling DC-SIGN targeted cross-presentation of exogenous peptides on MHC-I, are not completely resolved yet. In this paper, we used imaging flow cytometry and antigen-specific CD8^+^ T cells to investigate the intracellular fate of DC-SIGN and its cargo in human DCs. Our data demonstrates that immature DCs and toll-like receptor 4 (TLR4) stimulated DCs had similar internalization capacity and were both able to cross-present antigen targeted *via* DC-SIGN. Interestingly, simultaneous triggering of TLR4 and DC-SIGN on DCs resulted in the translocation of cargo to the cytosol, leading to proteasome-dependent processing and increased CD8^+^ T cell activation. Understanding the dynamics of DC-SIGN-mediated uptake and processing is essential for the design of optimal DC-SIGN-targeting vaccination strategies aimed at enhancing CD8^+^ T cell responses.

## Introduction

Dendritic cells (DCs) are antigen-presenting cells (APCs) that reside in all tissues and use germ-line encoded receptors to sample the tissue environment for pathogens. Upon pathogen recognition, DCs migrate to secondary lymphoid tissues, while they mature and process the internalized antigen to initiate antigen-specific T cells leading to humoral and/or cellular immune responses. Among the different receptors used by DCs to detect pathogens are C-type lectin receptors (CLRs), a large family of receptors that recognize carbohydrates in a Ca^2+^-dependent manner. Whereas some pattern-recognition receptors, such as toll-like receptors (TLRs), are specialized in activating intracellular signaling cascades to initiate DC maturation, CLRs primarily mediate pathogen endocytosis *via* internalization motifs present in their cytoplasmic domains (1, 2). This mechanism allows the efficient processing of pathogens for loading on MHC class II and I molecules and presentation to CD4^+^ and CD8^+^ T cells, respectively. These capacities of CLRs make them potent targets for vaccine development, especially for the induction of cellular responses for cancer treatment. The first studies on the targeting of CLRs have been done using DEC205-specific antibodies (Abs). These studies showed that targeting antigens to DCs resulted in prolonged and increased T cell responses when administered with an adjuvant. Also the amount of antigen needed for the induction of this response *in vivo* was considerately lower than when free antigen was used (3). The CLR DC immunoreceptor (DCIR) containing an immunoreceptor tyrosine-based inhibitory motif and present on a variety of blood and skin DC subsets, also mediated increased CD8^+^ T cells responses. This effect was further enhanced by the addition of a TLR 7/8 agonist (4). DC-SIGN is a type II membrane CLR discovered as a cell-adhesion receptor that supports primary immune responses (5) and enhances HIV infection of CD4^+^ T cells (6). DC-SIGN is expressed on monocyte-derived DCs (moDCs) in peripheral tissue, CD14^+^ dermal DCs in the dermal layers of the skin (7), and mature DCs in lymphoid tissues, however, DC-SIGN expression is lacking on follicular DCs and CD1a^+^ Langerhans cells (8). The carbohydrate recognition domain (CRD) of DC-SIGN contains a Ca^2+^-coordination site and has a dual specificity for high-mannose and Lewis-type carbohydrate structures (glycans), which gives the receptor the ability to recognize a broad variety of ligands (9), both on pathogens and self-glycoproteins (10). Lectin–glycan interactions have classically been considered to be of low affinity (11). As DC-SIGN is present in nano-clusters on the cell surface (12), the concept of avidity is of importance in the design of DC-SIGN-targeting compounds for *in vivo* vaccination strategies. We have explored the possibility of using DC-SIGN-targeting glycoconjugates for triggering of T cell responses (13–15) and demonstrated that DC-SIGN not only induces potent CD4^+^ T cell responses by targeting antigen to the endo-lysosomal pathway (16) but also triggers CD8^+^ T cell responses that can be boosted by supplementing a TLR4 stimulus. Unfortunately, the mechanism by which the intracellular routing initiated by DC-SIGN results in MHC-I presentation has not been fully identified. Understanding this mechanism will help in designing DC-SIGN-targeting vaccination strategies for the induction of anti-tumor immunity.

Dendritic cells are the most potent APC subset capable of priming naïve CD8^+^ T cells with exogenous antigen, for the induction of immunity against antigens derived from tumors or pathogens that do not infect DCs (17, 18). Although processing and presentation of endogenous proteins in MHC-II is quite well characterized, the mechanisms by which exogenous antigens are processed and loaded in MHC-I for presentation to CD8^+^ T cells (cross-presentation) are not fully understood. Cross-presentation efficiency and intracellular routing can differ depending on the mode of uptake, the antigen, and maturation status of the DC (19). To date two main routes of antigen cross-presentation have been described, namely the cytosolic and vacuolar pathway. In the vacuolar pathway, the exogenous antigens are processed by proteases and reloaded on MHC-I molecules without leaving the endosome. Cross-presentation *via* the vacuolar pathway has shown to be independent of TAP and degradation by the proteasome. By contrast, in the cytosolic pathway, the exogenous acquired antigens translocate to the cytosol and are processed by the proteasome, before they are loaded on MHC-I molecules. It remains elusive if loading of MHC-I is done by the endogenous MHC-I loading mechanism in the ER or by the possible recruitment of these MHC-I peptide loading complexes to phagosomes and endosomes (18, 19, 20).

Here, we used imaging flow cytometry to track DC-SIGN and its ligand in DCs and their co-localization with the different compartments involved in antigen processing and presentation. To further unravel the intracellular fate of the DC-SIGN ligand, we treated moDCs with different inhibitors of antigen processing. Our results demonstrate that DC-SIGN directs its cargo to early endosomal compartments, where the receptor–cargo complex partly dissociates. Since maturation status of the DCs can influence CD4^+^ and CD8^+^ T cell priming by means of co-stimulation and cytokine secretion, TLR agonists are often used as adjuvant to induce proper T cell responses. However, TLR stimulation can also influence antigen routing within the DCs, thereby changing the cross-presentation capacity (20). We observed that the cross-presentation capacity of DC-SIGN greatly depends on concomitant TLR4 triggering, which induces translocation of the ligand from the endosomes to the cytosol, where it can be efficiently routed for loading on MHC-I and subsequent CD8^+^ T cell activation.

## Materials and Methods

### Chemicals and Abs

The following reagents were used: *E. coli* lipopolysaccharide (LPS) (Sigma-Aldrich, MO, USA), monophosphoryl lipid A (MPLA) from *Salmonella enterica* (Invivogen), Paraformaldehyde (formaldehyde) aqueous solution (Electron Microscopy Sciences), Saponin (Sigma-Aldrich), and BSA (Roche). Abs used include: CD83-PE (Beckman Coulter), CD80-PE (clone L307.4, BD Biosciences), CD86-PE (clone 2331, BD Biosciences), EEA1-FITC (clone 14/EEA1, BD Biosciences), HLA-DM-PE (clone MaP.DM1, BD Biosciences), LAMP-FITC (clone H4A3, BD Biosciences), polyclonal rabbit-α-rab 11 (Invitrogen), HLA-A2-PE (BD Biosciences), CD107a-AF488 (BioLegend), CD107b-AF488 (BioLegend), Pacific Orange-labeled goat-α-rabbit IgG (Invitrogen), AF594-labeled goat-α-mouse IgG2a (Invitrogen), AF488-labeled goat-α-mouse IgG2b (Invitrogen), and biotin-labeled horse-α-mouse IgG (Vector Labs). CSRD (8), the polyclonal Ab against DC-SIGN, and AZN-D1 (5), a murine monoclonal IgG1 Ab against the carbohydrate recognition domain of DC-SIGN, were from our own stocks. DC-28 (21), the monoclonal IgG2a Ab against the stalk region of DC-SIGN was a kind gift of R. Doms (University of Pennsylvania). AZN-D1 was labeled with AF405 (Invitrogen) according to manufacturer’s instructions. AZN-D1 coated fluorescent beads were made as previously described (22). Gp100 with a C-terminal cysteine was conjugated to AZN-D1 *via* thiol-mediated conjugation using the bifunctional linker SMCC [succinimidyl 4-(*N*-maleimidomethyl)cyclohexane-1-carboxylate, Thermofisher Scientific, Breda]. Briefly, 5 mg AZN-D1 was activated with eight equivalents of SMCC in 50 mM phosphate buffer pH 8.3 containing 10 mM EDTA and 100 mM NaCl. After desalting the activated AZN-D1 over Sephadex-25 desalting columns (GE Healthcare Life Sciences, Breda), 12 equivalents of gp100 is added and vortexed thoroughly. The reaction is incubated for 1 h at 37°C. Final product is purified over Superdex 75 column (10 × 300, GE Healthcare Life Sciences, Breda).

### Cells

Monocytes were obtained from buffy coats of healthy donors, with informed consent (Sanquin, Amsterdam, reference: S03.0023-XT). Monocytes were isolated through a sequential Ficoll/Percoll gradient centrifugation (purity, >85%) and cultured in RPMI 1640 (Invitrogen) supplemented with 10% FCS (BioWhittaker), 1,000 U/ml penicillin (Lonza), 1 U/ml streptomycin (Lonza), and 2 mM glutamine (Lonza) in the presence of IL-4 (262.5 U/ml; Biosource) and GM-CSF (112.5 U/ml; Biosource) for 4–7 days (23). MoDC differentiation and maturation was monitored by FACS analysis (Calibur, Fortessa BD Biosciences) of DC-SIGN, CD83, CD80, and CD86. Stable CHO/DC-SIGN transfectants (24) were maintained in RPMI 1640 medium containing 10% FCS, 1,000 U/ml penicillin, 1,000 U/ml streptomycin, 2 mM glutamine, and 1 mg/ml geneticin (Invitrogen).

### Pulse-Chase Experiments

Approximately 10^6^ moDCs were incubated for 20 min in 100 µl of ice-cold culture medium. AF405-labeled AZN-D1 10 µg/ml was added and incubated for 30 min on ice. Cells were washed once with ice-cold medium and then transferred to 37°C for indicated time points or kept on ice. At indicated time points, cells were washed with ice-cold PBS, fixed in ice-cold 4% PFA in PBS for 20 min, and then washed two times with ice-cold PBS. For intracellular stainings, cells were permeabilized in 0.1% saponin in PBS for 30 min at room temperature and then blocked with a solution containing 0.1% saponin, 2% BSA, and 1% goat serum in PBS. Primary and secondary antibody stainings were performed in PBS with 0.1% saponin and 2% BSA at room temperature. After staining, cells were kept at 4°C in PBS supplemented with 0.5% BSA and 0.02% NaN_3_ until analysis.

### Antigen Presentation to Human CD8^+^ T-Cells

Immature moDCs were seeded in 96-well plates (Greiner) at 20 × 10^3^ cells/well and incubated with the different antigens in the presence or absence of the TLR4 ligand MPLA (10 µg/ml). After 3 h, cells were washed three times with RPMI and co-cultured overnight with a gp100_280–288_ TCR transduced CD8^+^ HLA-A2 restricted T cell clone (25) (10^5^ cells per well, E:T ratio 1:5). IFNγ in the supernatant was measured by sandwich ELISA according to protocol (Biosource). To determine the effect of proteasomal and endosomal inhibitors, moDCs (30 × 10^3^ cells/well) were incubated with chloroquine (25 µM, Sigma), MG132 (10 µM, Selleck), epoxomicin (0.25 µM, Selleck), or cathepsin S inhibitor (5 µM, calbiochem) at 37°C for 30 min prior to the addition of antigen and the TLR4 ligand LPS (100 ng/ml). After 3 h, the moDCs were washed and co-cultured with a gp100_280–288_ TCR transduced CD8^+^ HLA-A2 restricted T cell clone (10^4^ cells per well, E:T ratio 3:1) (25). Degranulation was analyzed by flow cytometry, *via* the membrane staining of CD107a and CD107b, as a measure for T cell activation.

### Confocal Laser-Scanning Microscopy (CLSM)

Stained cells were allowed to adhere to poly-l-lysine-coated glass slides and mounted with anti-bleach reagent vinol. Samples were analyzed using a 63×/1.4 HCX PL APO CS oil objective on a TCS SP2 AOBS confocal microscope (Leica Microsystems GmbH). Images were acquired using LCS 2.61 (Leica Microsystems GmbH) and processed using Adobe Photoshop CS4 or ImageJ.

### Live Cell Imaging

CHO/DC-SIGN cells were cultured on gelatin coated glass slides. AZN-D1 coated beads were added to the cells and followed for different time points. Cells were analyzed by means of a 3I Marianas™ digital imaging microscopy workstation (Zeiss Axiovert 200 M inverted microscope Carl Zeiss), equipped with a nanostepper motor (*Z*-axis increments 10 nm) and a cooled CCD camera (Cooke Sensicam, 1,280 × 1,024 pixels Cooke Co). Visualization was performed with a 40× air lens. The microscope, camera, and data viewing process were controlled by SlideBook™ software (version 4.0.8.1 Intelligent Imaging Innovations).

### Imaging Flow Cytometry

Cells were acquired on the ImageStreamX (Amnis corp.) imaging flow-cytometer. A minimum of 15 × 10^3^ cells was acquired per sample at 40× magnification at a flow rate ranging between 50 and 100 cells/s. Analysis was performed using the IDEAS v6.0 software (Amnis corp.). A compensation table was generated using the compensation macro built in the software and applied to the single staining controls. Proper compensation was then verified by visualizing samples in bivariate fluorescence intensity plots (Figure S1 in Supplementary Material). A template analysis file to gate for single optimally focused cells (Figure S2 in Supplementary Material) and applied to the experimental samples in order to export this population to a new compensated image file to allow merging all experimental samples within a single file for direct sample analysis. Ag/receptor internalization was investigated using a combination of a mask designed to detect the intracellular space and the internalization feature (Figure S3 in Supplementary Material).

Co-localization was calculated using the bright detail similarity R3 feature on a whole cell mask. Co-localization is calculated as the logarithmic transformation of Pearson’s correlation coefficient of the localized bright spots with a radius of 3 pixels or less within the whole cell area in the two input images (bright detail similarity R3). Since the bright spots in the two images are either correlated (in the same spatial location) or uncorrelated (in different spatial locations), the correlation coefficient varies between 0 (uncorrelated) and 1 (perfect correlation). The logarithmic transformation of the correlation coefficient allows the use of a wider range for the co-localization score. In general, cells with a low degree of co-localization or no co-localization at all between two probes show scores below 1.

### mRNA Isolation, cDNA Synthesis, and Real-Time PCR

After cell lysis, mRNA was isolated by mRNA Capture kit (Roche) and cDNA was synthesized with the Reverse Transcription System kit (Promega) following manufacturer’s guidelines. cDNA was diluted 1:2 in nuclease-free water and stored at −20°C until analysis. Primers specific for human DC-SIGN (5′-aacagctgagaggccttgga-3′, 5′-gggaccatggccaagaca-3′) and GAPDH (26) were designed with Primer Express 2.0 (Applied Biosystems) and synthesized at Invitrogen (Invitrogen). Primer specificity was computer-tested (BLAST, National Center for Biotechnology Information) and confirmed by dissociation curve analysis. Real-time PCR reactions were performed using the SYBR Green method in an ABI 7900HT sequence detection system (Applied Biosystems) as previously described (26).

### Statistics

Unless otherwise stated, data are presented as the mean ± SD of at least three independent experiments. Statistical analyses were performed using the statistical package SPSS. Statistical significance was set at *P* < 0.05 and it was evaluated by the Mann–Whitney *U* test.

## Results

### DC-SIGN Is Exclusively Localized at the Cell Membrane and Is Quickly Internalized Upon Receptor Ligation

**Figure 1:**
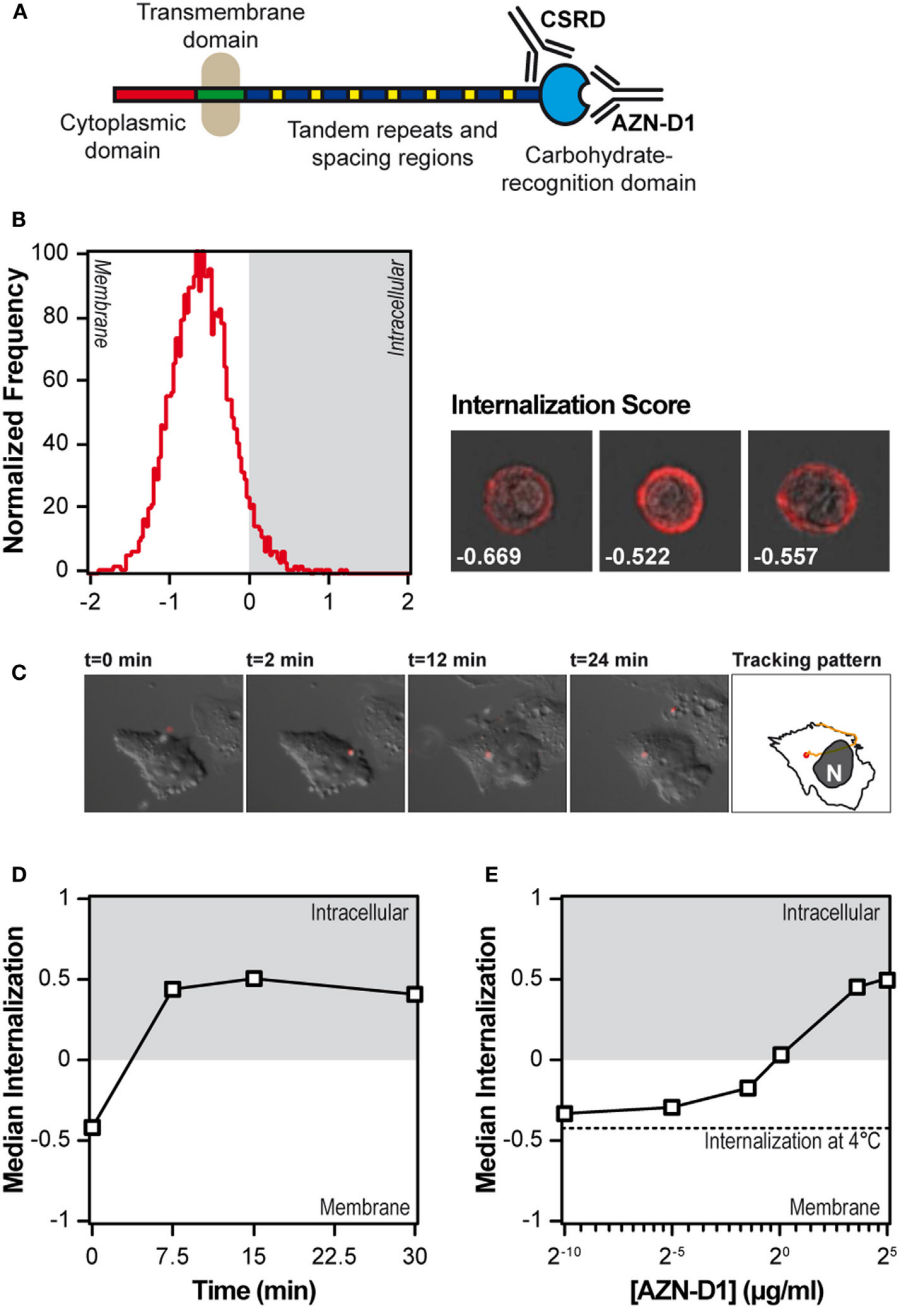
DC-SIGN on immature monocyte-derived DCs (moDCs) is exclusively expressed at the extracellular membrane and quickly internalizes upon triggering. **(A)** Summary of the anti-DC-SIGN antibodies used in the present study. **(B)** Internalization score of resting immature moDCs, after fixation, permeabilization and staining with a polyclonal antibody against DC-SIGN (*n* > 5,000). Next to the histogram, three representative images are included with their respective internalization score. **(C)** Still frames of a live cell imaging experiment in which DC-SIGN-CHO cells were exposed to AZN-D1-coated fluorescent beads. The right-most frame shows the tracking pattern, representative of eight experiments. **(D)** Time-course of the median internalization score of moDCs triggered with AF405-labeled AZN-D1 (*n* > 5,000). **(E)** Effect of the AF405-labeled AZN-D1 concentration on the internalization score after 30 min at 37°C. The dotted line indicates the internalization of the highest antibody concentration after 30 min at 4°C (*n* > 5,000).

We first tested the steady state localization of DC-SIGN on moDCs using imaging flow cytometry, a technology that allows for the quantification of morphological aspects of images acquired from large populations of cells. The localization of DC-SIGN on fixed moDCs was assessed *via* staining with the anti-DC-SIGN polyclonal Ab CSRD (8), which does not interfere with the carbohydrate-binding site of DC-SIGN (see Figure 1A). An internalization score higher than 0 indicates that the fluorescent signal is mainly localized inside the cell, whereas a negative internalization scores reflects exclusive membrane localization. When the intracellular and membrane localization are equal, the internalization score is set to 0. In the steady state, DC-SIGN in DCs is exclusively expressed on the cell membrane (Figure 1B), since more than 95% of the moDCs had a negative internalization score. We confirmed the exclusive membrane localization of DC-SIGN, using the left over cells from the imaging flow cytometry for CLSM imaging (Figure S4 in Supplementary Material). To investigate the kinetics of internalization, DC-SIGN was stably transfected in CHO cells, exposed to AZN-D1-coated fluorescent beads and followed by live cell widefield epifluorescence imaging. AZN-D1 is a monoclonal Ab against the carbohydrate-binding site of DC-SIGN and is known to trigger receptor internalization (see Figure 1A) (16). The still frames in Figure 1C show how a bead adheres to the surface of the cell within seconds and is quickly internalized, approximately 2 min after receptor ligation.

Because the mechanisms of internalization of particulate and soluble antigen may vary, we also investigated the internalization of AF405-labeled AZN-D1. First, moDCs were incubated in the presence of AF405-labeled AZN-D1 for 30 min at 4°C. Then the cells were transferred to 37°C for the indicated time points, washed, fixed, and analyzed by imaging flow cytometry. The maximum level of AZN-D1 internalization was already achieved at 7.5 min (Figure 1D), indicating that DC-SIGN internalization is a fast process. To investigate whether receptor internalization was dependent on the amount of antigen available, we repeated the pulse-chase experiment with a titration of AF405-labeled AZN-D1 and then fixed, permeabilized, and stained the receptor with CSRD (8). At 1 µg/ml, the amount of internalized receptor equaled the amount of receptor on the surface (internalization score 0) and total internalization was achieved using 5 µg/ml of ligand (Figure 1E).

**Figure 2:**
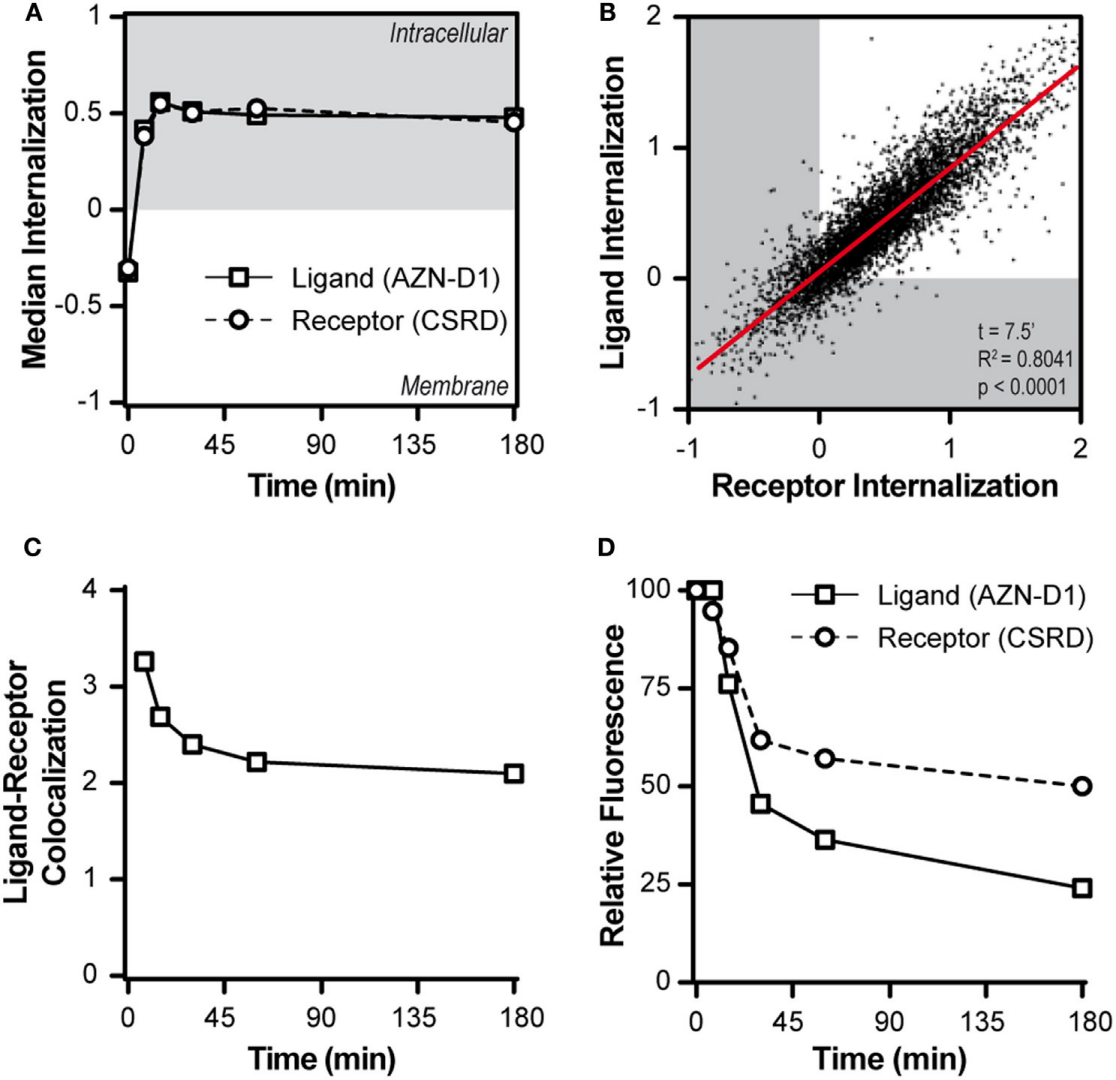
DC-SIGN and its cargo quickly dissociate upon internalization. **(A)** Time-course of the median internalization score of monocyte-derived DCs triggered with AF405-labeled AZN-D1 and stained intracellular against DC-SIGN (*n* > 5,000). **(B)** Scatter plot of the internalization scores of both ligand and receptor 7.5 min after triggering with AF405-labeled AZN-D1 (*n* > 5,000). **(C)** Time-course of the median co-localization of AF405-labeled AZN-D1 and DC-SIGN (*n* > 5,000). **(D)** Time-course of the fluorescence signal intensity of both AF405-labeled AZN-D1 and DC-SIGN (*n* > 5,000).

Subsequently, we tracked both ligand and receptor in a time-course pulse-chase experiment using the AF405-labeled AZN-D1 Ab to model the ligand and staining with CSRD after fixation and permeabilization to track the receptor. Upon DC-SIGN triggering, both ligand and receptor were quickly internalized and DC-SIGN did not return to the membrane after internalization (Figure 2A). At an early time point (7.5 min), the internalization of receptor and ligand almost perfectly correlated, implying an interdependence of both processes (Figure 2B). When the co-localization score of the ligand and the receptor was assessed, we observed that the co-localization score was maximal at baseline (*t* = 0 min) and decreased very quickly once both ligand and receptor were internalized (Figure 2C), indicating that ligand and receptor partly dissociate. We also assessed the amount of ligand and receptor at the different time points during the experiment. The signal for the ligand decayed by almost 80% during the experiment (Figure 2D). By blocking vesicular degradation with chloroquine, we were able to significantly reduce ligand decay after 30 min (Figure S5 in Supplementary Material), indicating that the ligand gets (partly) processed in the endosomes. We stained for the receptor after fixation and permeabilization of the cells, allowing us to detect the total amount of intracellular and membrane associated DC-SIGN. We observed a reduction in receptor signal to approximately 50–60% of the starting amount (Figure 2D). The loss of signal indicates that DC-SIGN gets degraded and does not recycle to the membrane. This is supported by previous work of Tacken et al. (27) showing that in the presence of the protein synthesis inhibitor cycloheximide barely any newly synthesized DC-SIGN molecules re-emerged on the cell surface within 3 h following DC-SIGN mediated internalization. Even when DC-SIGN was targeted for a prolonged period of time, the surface expression of DC-SIGN was significantly decreased up to 2 days after Ab removal. Taken into account that the recycling of receptors is a fast process that often takes places within minutes after receptor internalization our results suggest that DC-SIGN is slowly degraded and not recycled, while the ligand of DC-SIGN quickly gets processed after internalization.

### DC-SIGN Directs Its Cargo Through Early Endosomal Compartments Before Dissociation

**Figure 3:**
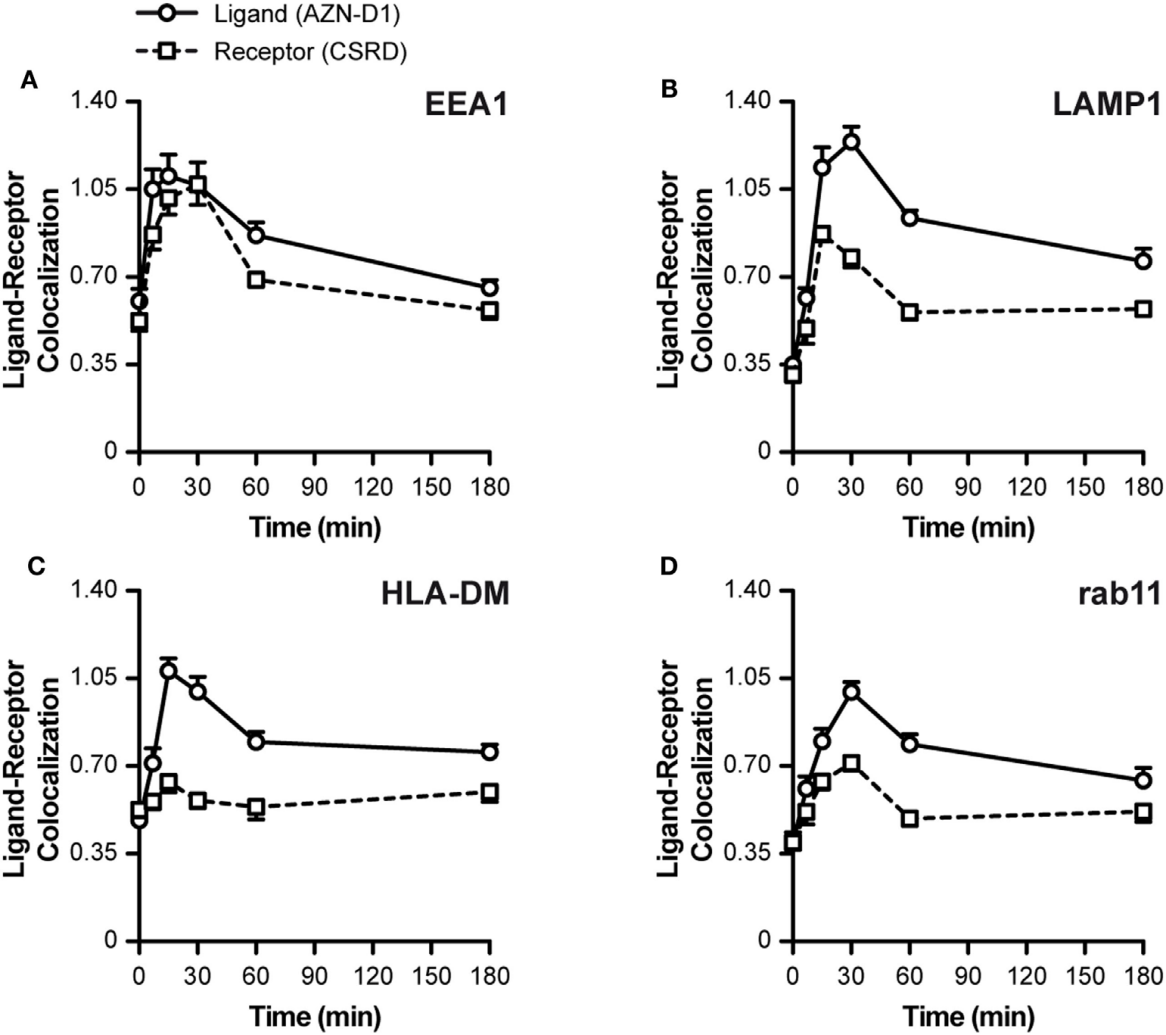
Intracellular routing of DC-SIGN and its ligand. Monocyte-derived DCs were pulsed with AF405-labeled AZN-D1 for 30 min on ice and transferred to 37°C. Cells were fixed at indicated time points and stained with the CSRD antibody to localize DC-SIGN. Time-course of the co-localization scores of AF405-labeled AZN-D1 (mean ± SEM) and CSRD (DC-SIGN) with **(A)** EEA1, **(B)** LAMP1, **(C)** HLA-DM, and **(D)** Rab11 (*n* > 5,000).

To investigate the fate of both ligand and receptor, we measured the co-localization scores of both ligand (AZN-D1) and receptor (CSRD) (see Figure 1A) with Abs commonly used to track endocytic compartments. Until approximately 30 min both ligand and receptor co-localized evenly with the early endosomal marker EEA1 (both scores around 1.05). Hereafter, the co-localization for the receptor dramatically decreased, whereas co-localization with the ligand slowly decreased, suggesting that ligand and receptor partly dissociate in early endosomes (Figure 3A). This is further supported by the LAMP1 (lysosomes) co-localization scores, which show that the ligand (but not DC-SIGN receptor) reached the lysosomes before 30 min, while peaking at around 45 min (Figure 3B). In accordance, the MHC-II compartment co-localized with the ligand (at 30 min score 1.05), but not with the receptor (30–180 min score 0.6, Figure 3C). Interestingly, rab11 shows a moderate co-localization with the ligand (30 min score 1.05), but a poor co-localization with the receptor (score 0.6), suggesting that routing to this compartment is receptor-independent and may follow upon a stay at the early endosomes or the lysosomes (Figure 3D). The decay observed for the receptor in Figure 2D might be explained by quick lysosomal degradation, but since there is very little co-localization of the receptor with the lysosomal marker LAMP1, degradation could also already occur in the early endosomes.

Our data indicate an internalization model in which DC-SIGN mediates the internalization of its antigen ligand, which ends up in early endosomes where the receptor–ligand complex dissociates. The released cargo continues its way to lysosomes by the maturation of early endosomes, while a fraction of receptor–ligand complexes possibly translocate to the cytosol to initiate MHC-I loading.

### Simultaneous DC-SIGN and TLR4 Triggering Affects DC-SIGN Internalization

**Figure 4:**
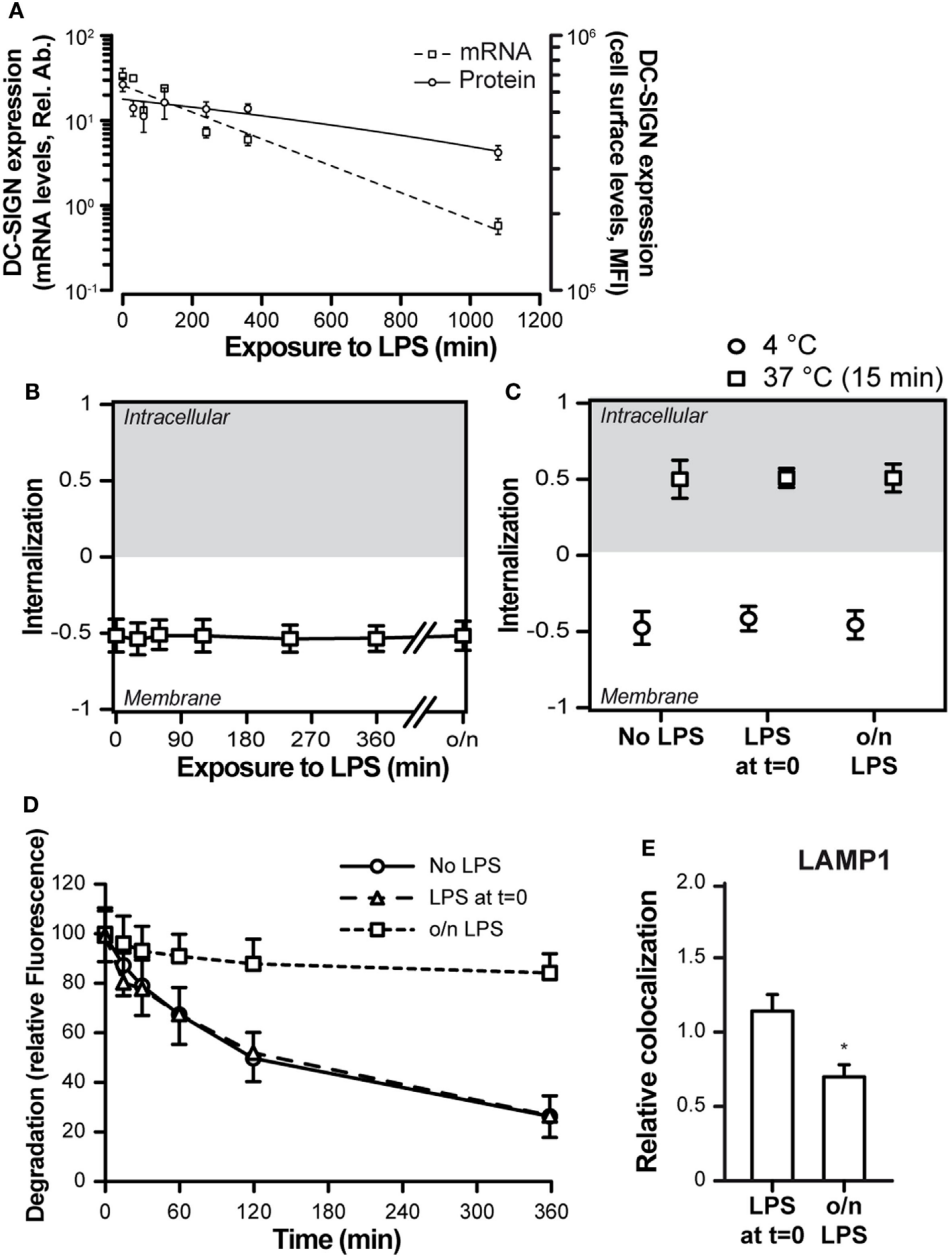
Toll-like receptor 4 (TLR4) triggering affects the routing of DC-SIGN and its ligand. **(A)** Time-course of the expression levels of DC-SIGN at both the mRNA and protein level after TLR4 [lipopolysaccharide (LPS)] stimulation of monocyte-derived DCs. **(B)** Time-course of the internalization score of DC-SIGN after treatment with a TLR4 ligand (LPS). **(C)** Internalization score of AF405-labeled AZN-D1 after a 60-min incubation at 4°C or a 15-min incubation at 37°C. **(D)** Time-course of the fluorescence signal intensity of AF405-labeled AZN-D1 **(E)** Co-localization scores (relative to no LPS) of AF405-labeled AZN-D1 with LAMP1, 60 min after triggering. Mean ± SEM (*n* > 5,000). *P < 0.01 compared to no LPS.

TLR4 triggering is commonly used to address the effects of DC activation and maturation, a process that typically occurs upon pathogen recognition and that is necessary for proper antigen processing, presentation, and CD8^+^ T cell priming (28). In addition, DC-SIGN triggering has been described to elicit a signaling cascade that modulates the TLR4-induced signaling (29, 30). We therefore investigated the consequences of DC maturation on DC-SIGN internalization. First, we investigated the effect of TLR4-mediated moDC activation on DC-SIGN expression levels. LPS treatment of moDCs resulted in a dramatic decrease in both DC-SIGN protein (10-fold) and mRNA (100-fold) after 18 h (Figure 4A). The decrease in DC-SIGN expression was not accompanied by an internalization of the receptor, as it was still located on the cell membrane (Figure 4B), indicating that DC-SIGN was lost by either shedding into the supernatant or by incorporation into exosomes, possibilities that have been previously described for DC-SIGN (31, 32). Still, simultaneous triggering of DC-SIGN and TLR4 (LPS at *t* = 0) or triggering of DC-SIGN on mature moDCs (overnight LPS treatment) had no consequences for the overall internalization rate, which proceeded as efficiently on mature moDCs as on immature moDCs (Figure 4C). Nevertheless, the fate of AZN-D1 ligand differed greatly between the simultaneous triggering of DC-SIGN and TLR4 (LPS at *t* = 0) and the triggering of DC-SIGN on matured moDCs (o/n LPS) (Figure 4D). While ligand degradation was similar in immature moDCs and moDCs that received LPS at *t* = 0, triggering of DC-SIGN on mature moDCs showed decreased ligand degradation. Less than 20% AZN-D1 degradation occurred in mature moDCs even after an extended incubation time (6 h), compared to 70–80% ligand degradation in immature moDCs and moDCs receiving LPS at *t* = 0 (Figure 4D). This was consistent with a reduced trafficking of AZN-D1 to the lysosomes upon overnight (o/n) treatment with LPS (Figure 4E).

### Simultaneous Triggering of DC-SIGN and TLR4 Affects the Cross-Presentation Route in DCs

**Figure 5:**
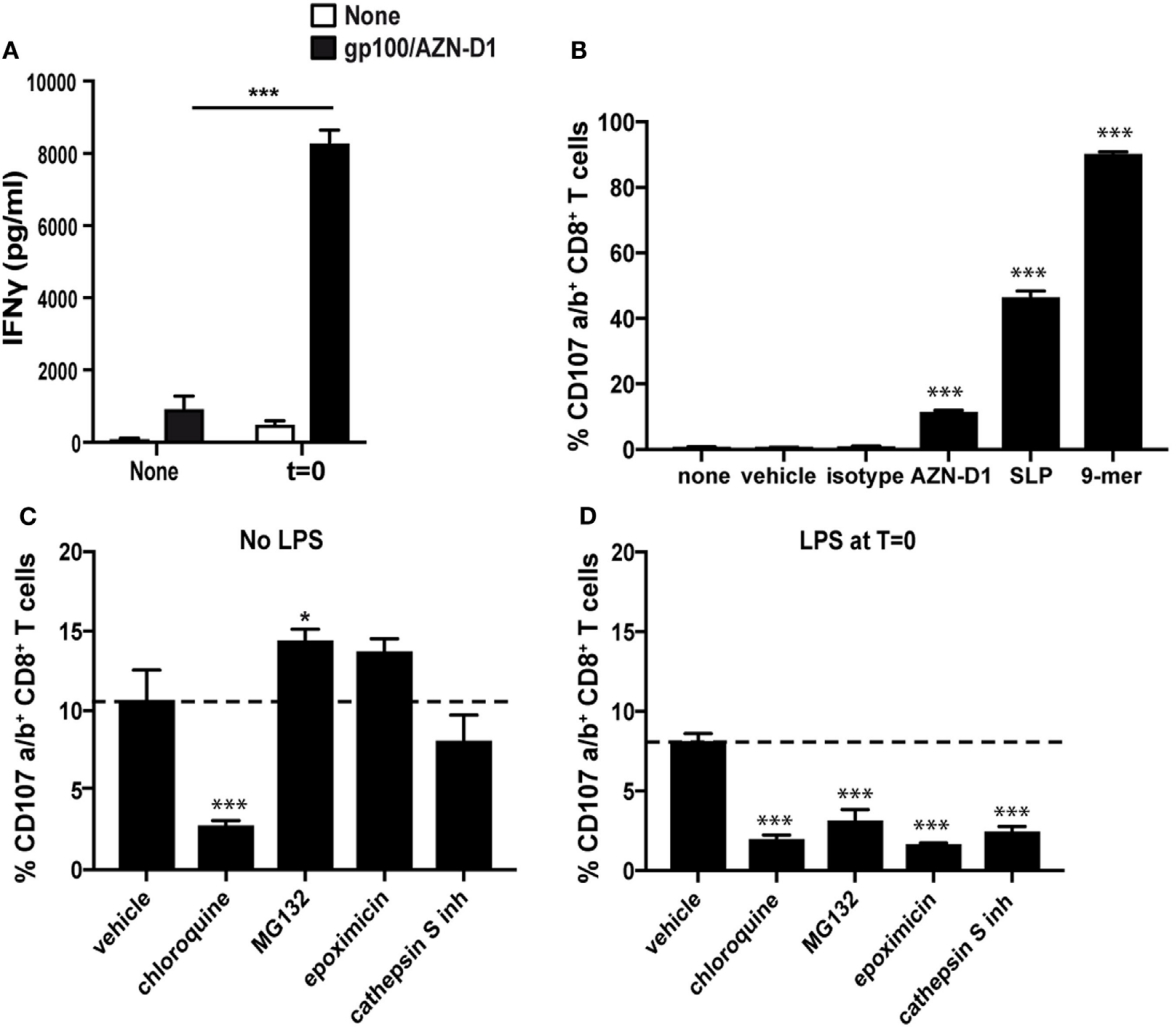
Toll-like receptor 4 (TLR4) triggering facilitates antigen translocation to the cytosol. **(A)** Immature monocyte-derived DCs (moDCs) and moDCs that received a TLR4 stimulus at *t* = 0 [monophosphoryl lipid A (MPLA)] were pulsed with gp100/AZN-D1 for 3 h and subsequently co-cultured o/n with gp100_280–288_ CD8^+^ T cells. IFNγ secretion was analyzed by ELISA as a measure for T cell activation. **(B)** Immature moDCs were incubated with 0.1% DMSO (vehicle), gp100/AZN-D1 (10 µg/ml), gp100 synthetic long peptide (SLP) (10 µM), and the gp100_280–288_ 9-mer minimal epitope (1 µg/ml) for 3 h. Thereafter, moDCs were co-cultured with gp100_280–288_ CD8^+^ T for 45 min and CD107a/b expression on the cell surface was analyzed as a measure for CD8^+^ T cell activation. Groups are significantly different compared to none, vehicle, and isotype. **(C)** Immature moDCs and moDCs that received a TLR4 stimulus [lipopolysaccharide (LPS)] at *t* = 0 **(D)** were incubated 30 min prior and during the 3 h antigen (gp100/AZN-D1) pulse with 0.1% DMSO (vehicle), chloroquine (25 µM), MG132 (10 µM), epoxomicin (0.25 µM), and cathepsin S inhibitor (5 µM). Groups are significantly different compared to AZN-D1. Data represented in mean ± SD, one-way ANOVA was performed, experiments are representative of a *N* = 2 for graph A and B and a *N* = 3 for graph C and D (*P < 0.05, ***P < 0.001).

To evaluate the effect of TLR4 triggering on cross-presentation after DC-SIGN targeting, we compared the capacity of antigen pulsed immature moDCs and TLR4-stimulated moDCs (*t* = 0), to activate CD8^+^ T cells. We excluded the DCs that were incubated o/n with a TLR4 stimulus, because of their greatly reduced DC-SIGN receptor surface expression. Therefore, pre-treatment with a TLR4 stimulus before antigen administration is not a favorable vaccine strategy when targeting DC-SIGN. As an antigen, we used a gp100 synthetic long peptide (SLP) (29-mer, VTHTYLEPGPVTANRQLYPEWTEAQRLDC) containing both the gp100_280–288_ CD8^+^ and gp100_45–59_ CD4^+^ T cell epitope conjugated to the DC-SIGN-targeting monoclonal antibody AZN-D1. A 3 h antigen pulse was followed by co-culturing of moDCs o/n with gp100_280–288_ specific CD8^+^ T cells, after which the released IFNγ was determined as a measure for T cell activation (Figure 5A). MoDCs that received TLR4 stimulus at *t* = 0 outperformed the immature moDCs in their capacity to activate CD8^+^ T cells. Next, we determined the specificity of our DC-SIGN targeting Ab by pulsing immature DCs for 3 h with gp100/AZN-D1 conjugates, gp100/mIgG1 isotype control conjugates (functioning as a negative control), the 29-mer gp100 SLP, and the 9-mer minimal epitope that can directly bind to MHC-I. After a 3 h antigen pulse, gp100_280–288_ specific CD8^+^ T cells were added to the moDCs for 45 min and stained for degranulation markers (CD107a and CD107b). MoDCs pulsed with the gp100/AZN-D1 were able to activate antigen-specific CD8^+^ T cells as measured by degranulation levels. By contrast, the gp100/mIgG1 conjugate induced no CD8^+^ T cell activation. The SLP as a single non-targeted agent induced degranulation of more than 40% of the CD8^+^ T cells, confirming the robustness of this assay (Figure 5B; Figure S6 in Supplementary Material). Therefore, the lower response induced by gp100/AZN-D1 is due to the limited amount of SLPs that can be conjugated to the antibody, rather than the sensitivity of the experiment.

The enhanced cross-presentation after TLR stimulation has been described to result from the induction of a different antigen-processing route (20, 33–36). To investigate if changes in DC-SIGN ligand routing after TLR4 stimulation is responsible for the observed increase in cross-presentation, we investigated the antigen-processing route by looking at CD8^+^ T cell activation after DC antigen loading in the presence of relevant inhibitors. For this experiment, we incubated immature and LPS-stimulated moDCs with chloroquine for blocking of endosomal acidification, cathepsin S inhibitors to block endosomal antigen-processing or MG132 and epoxomicin to inhibit proteasomal degradation of antigens. Treatment with the inhibitors mentioned above showed only minor differences in viability (Figure S7 in Supplementary Material). Interestingly, the routing of antigen in immature and LPS-treated moDCs differed substantially (Figures 5C,D). While the activation of CD8^+^ T cells by immature moDCs was not affected by the proteasome inhibitors MG132 and epoxomicin, LPS stimulated moDCs showed decreased CD8^+^ T cell activation in the presence of these inhibitors. Also inhibition of the protease cathepsin S reduced cross-presentation by LPS-stimulated moDCs, while it did not affect cross-presentation of DC-SIGN targeted antigens in immature moDCs. This indicates that DC-SIGN-mediated uptake and proteolysis of antigen in the endosomes/lysosomes of immature moDCs is not dependent on the protease cathepsin S. By contrast, chloroquine, a drug that inhibits acidification of endosomes, significantly reduced CD8^+^ T activation by both immature and LPS-stimulated moDCs. When we checked for HLA-A2 molecules on the cell surface after the inhibitor treatment, we observed a decrease of HLA-A2 expression on chloroquine treated moDCs, while the other inhibitors did not affect HLA-A2 expression (Figure S8 in Supplementary Material). However, the external loading of membrane expressed MHC-I molecules with the 9-mer minimal epitope in the presence of inhibitors did not result in a decrease in CD8^+^ T cell activation (Figure S9 in Supplementary Material). Therefore it is difficult to say if the observed decrease in CD8^+^ T cell activation after chloroquine treatment is related to the lower expression of HLA-A2.

Surprisingly, we did not observe any enhanced cross-presentation of TLR4-stimulated moDCs within the time frame of the degranulation assay (Figure S10 in Supplementary Material), which could possibly be explained by the short time window of antigen processing after the pulse. These results were further validated by measuring IFNγ secretion after an o/n culture with the gp100 specific CD8^+^ T cells (Figure S11 in Supplementary Material), confirming the enhanced CD8^+^ T cell activation by combining DC-SIGN targeting with a TLR4 stimulus. We observed a smaller inhibitory effect of the cathepsin S inhibitor on CD8^+^ T cell activation. This can be explained by the different time points and assays used to analyze the amount of CD8^+^ T cell activation. The secretion of IFNγ was measured in the supernatant after a co-culture of 16 h, while the percentage of degranulation was analyzed after a 45-min co-culture. In both experiments, the cells were treated with the inhibitors during the 3 h antigen pulse. Thereafter, cells were washed and co-cultured with the CD8^+^ T cells. Some of the inhibitors are reversible; therefore, the effect can decrease overtime explaining the difference in inhibitory capacity of the cathepsin S inhibitor (Figure S11 in Supplementary Material).

Together our results indicate that the combination of DC-SIGN targeting and TLR4 triggering leads to the escape of antigen to the cytosol, where it is further processed *via* the proteasome for cross-presentation.

## Discussion

In this study, we investigated the intracellular routing of the CLR DC-SIGN and its involvement in loading antigens on MHC-I through cross-presentation. While DC-SIGN targeting of antigen leads to cross-presentation by both immature and TLR4-stimulated DCs, we found a major contribution for TLR4 signaling, instigating an alternative intracellular cross-presentation route *via* the cytosol, which resulted in an increased capacity of moDCs to activate CD8^+^ T cells.

Targeting DC-SIGN with antigen conjugated Abs, glycan conjugated antigens or HIV virus, a natural ligand of DC-SIGN, results in efficient MHC-II and MHC-I loading and CD4^+^ T cell and CD8^+^ T cell activation, respectively (15, 16, 37–39). This makes DC-SIGN an attractive candidate for vaccine targeting strategies. Since vaccination strategies also rely on adjuvants inducing DC maturation, such as TLR agonists, understanding the intracellular fate of DC-SIGN and its ligand in both immature and TLR stimulated DCs is vital for vaccine development.

Previous studies have shown that pathogens and AZN-D1, both binding to the CRD, are taken up in a clathrin-dependent manner (40, 41), while DC-SIGN targeting *via* the neck region results in clathrin-independent internalization (42). Our data demonstrates that upon targeting the CRD with AZN-D1, DC-SIGN on immature moDCs is internalized within minutes and directed to early endosomes. At this stage, part of the DC-SIGN–ligand complexes begin to dissociate and proceed to late endosomes and lysosomes. Co-localization of the DC-SIGN ligand with the receptor was decreased after 15–30 min, which was followed in parallel with an increase of the antigen in the lysosomes. This indicates that once DC-SIGN ligands are dissociated from the receptor in the early endosomes, they are at least partly routed to maturing endosomes. Interestingly, the dissociation between the ligand and receptor occurs at the maturing endosomes, indicating that ligand and receptor follow different intracellular routes. Although DC-SIGN does not return to the membrane, we were not able to clarify its intracellular fate upon release from its ligand. Nevertheless, the clear fluorescence signal decay and previous work showing that prolonged DC-SIGN targeting with AZN-D1 significantly reduced the surface expression for up to 48 h (27) suggests that DC-SIGN is targeted for destruction. Since we observed that DC-SIGN poorly co-localizes with the lysosomes, we hypothesize that it is degraded by a different mechanism, which has not yet been identified. Endocytosis *via* DC-SIGN is regulated by a dileucine (LL) motif in the cytoplasmic tail of the receptor (16, 43) which might function as potential targets for ubiquitination. Different modes of ubiquitination exist that regulate among other protein degradation (44). Polyubiquination of the C-type lectin Mannose receptor facilitates antigen translocation from the endosomes to the cytosol (45), indicating that this process of receptor ubiquitination is a recognized mechanism, whereby CLRs redirect their cargo to the cytosol. Possibly DC-SIGN also uses this mode of action.

Our data shows an important role for the timing of the maturation stimulus when targeting antigens *via* DC-SIGN for cross-presentation to CD8^+^ T cells. Triggering of TLR4 has been described to lead to an enhanced cross-presentation of soluble antigen until approximately 16 h after stimulation, while fully matured DCs (LPS for >24 h) showed a decreased ability to cross-present antigen (20, 33). Apparently, 16 or 24 h of LPS stimulation can make a major difference for the ability of DCs to efficiently cross-present antigen. In our experimental setup, moDCs were stimulated with LPS o/n (16 h) and therefore should still be in the enhanced cross-presentation phase. In fact, we saw decreased shuttling of the ligand to LAMP1 positive compartments in line with the findings of Alloatti et al., who showed a decreased phago-lysosomal fusion after TLR triggering, resulting in decreased degradation, thereby supporting cross-presentation (33). While LPS did not affect the uptake capacity of the DC-SIGN receptor, its expression was dramatically decreased on both mRNA and protein level, which would result in an overall decrease in antigen uptake. Based on these data, administration of the adjuvant before providing the antigen *via* DC-SIGN targeting would not be a favorable vaccination strategy. Multiple studies have described that the enhanced efficiency of cross-presentation after TLR triggering is due to a change in antigen routing and processing, like enhanced translocation to the cytosol and increased activity of the proteasome (34–36). To investigate if a different route of antigen processing was responsible for the increase in cross-presentation, we blocked different molecules known to be important for MHC-I and MHC-II presentation in immature DCs and DCs that received a TLR4 stimulus at *t* = 0. We observed a striking difference in antigen routing as early as 3 h after antigen pulse. Both the immature and TLR4-stimulated moDCs were sensitive to chloroquine, a drug that inhibits endosomal acidification. Unexpectedly, chloroquine had a reducing effect on MHC-I expression, making it difficult to conclude if the observed effect on CD8^+^ T cell activation is due to the inhibition of cross-presentation or to the reduced expression of MHC-I on the cell surface. MoDCs that received a TLR4 trigger at *t* = 0 showed a significant dependence on proteasome activity, a mechanism not observed in immature DCs. Thus, our results suggest that following TLR4 triggering antigens translocate from the endosome to the cytosol, thereby entering the cytosolic pathway of cross-presentation. This route of MHC-I loading also has been described for natural DC-SIGN ligands (HIV-1 virions) (39). It has been described that TLR triggering can result in antigen translocation from endosomes to the cytosol. Dingjan et al. showed that upon LPS triggering the NOX2 complex in phagosomes produces reactive oxygen species resulting in lipid peroxidation, thereby inducing membrane damage and the release of antigen from these “leaky” endosomes (35). This was a rather quick process, already observed 30 min after LPS stimulation. Also a role for sec61 in the endosomal escape after TLR triggering has been reported (36). Our results stress the importance of appropriately timing the maturation stimulus when targeting antigens to DC-SIGN, as not only the antigen enters a more efficient route of cross-presentation, but also the fate of the DC-SIGN receptor is dependent on maturation status of the DC.

The recycling endosome is characterized, among others, by Rab11, which allows direct recycling to the plasma membrane, but also to the secretory pathway through the trans-Golgi network (46). Our data show that co-localization of the DC-SIGN ligand with Rab11 follows the same pattern as HLA-DM, a molecule associated with the MHC-II loading compartment. Since we cannot observe the return of DC-SIGN ligands to the plasma membrane and the MHC-II compartment originates from the trans-Golgi network, it is likely that Rab11 facilitates a connection between early endosomes and the MHC-II loading compartment without further contribution of lysosomal degradation.

The regulation of the internalization and intracellular routing of DC-SIGN on DCs is an important aspect for the rational design of antibody and glycan-based DC-SIGN-targeting vaccines (47). Based on this study, the use of DC-SIGN ligands in combination with TLR4 ligands would serve as excellent antigen targeting platforms to enhance the antigen cross-presentation in DC-based anti-tumor vaccination strategies.

## Author Contributions

All the authors were responsible for design of the experiments; data collection was performed by SH, SD, KB, PS, RM, and JG-V; all authors participated in the data analysis and interpretation; SH, SD, KB, SV, JG-V, and YK drafted and critically revised the article; all authors gave their approval for publication.

## Conflict of Interest Statement

The authors declare that the research was conducted in the absence of any commercial or financial relationships that could be construed as a potential conflict of interest.

## Supplementary Material

Figure S1 Monocyte-derived DCs (moDCs) were fixed and measured by imaging flow cytometry. Once the compensation table was calculated for each of the staining sets, it was applied to the single staining samples that were acquired using the same settings as experimental samples. Proper compensation was then verified by visualizing samples in bivariate fluorescence intensity plots. Representative images are displayed underneath the corresponding dotplots.

Figure S2 **(A)** After the application of the compensation table, cells were plotted in an area vs aspect ratio intensity bivariate scatter plot. Several populations could be identified. Population 1 was characterized by small area and high aspect ratio intensity. Images from the population 1 gate clearly show the events correspond to beads. Population 2 had an average area of approximately 100 square pixels and high aspect ratio intensity. Images from the population 2 gate show that these cells are small single cells with a large nucleus, suggesting these cells could be lymphocytes, a common contamination in Percoll-isolated monocyte-derived cell cultures. Population 3 had an area between 150 and 300 square pixels and an aspect ratio intensity higher than 0.6. These cells, the biggest population, represent dendritic cells in single cell suspension. The remaining populations (4 and 5) had a larger area and/or low aspect ratio intensity, suggestive of cell doublets and aggregates, as demonstrated in the corresponding imagery. **(B)** Gradient RMS on the brightfield channel 1 shows that the majority of the cells had a sharp contrast. Images have been selected with gradient RMS values across the whole range of gradient RMS values of the population. The threshold can then be manually set up in approximately 60.

Figure S3 **(A)** First, a morphology mask is applied to the brightfield channel (channel 1). This mask takes the whole perimeter of the cell. Then, 5 pixels are eroded from this mask until the membrane of the cell is left out of the mask. The resulting mask is applied to the channel containing the probe of interest and a ratio of the intensity inside the mask relative to the total intensity of the cell is calculated. **(B)** Monocyte-derived DCs exposed to AZN-D1 for 30 min at 4°C show a membrane-bound pattern of staining, with a median internalization score of −0.985. When these cells are incubated at 37°C for 2 h, the probe is internalized and the internalization score increases to 1.002. A selection of cells with internalization scores ranging from −1 to 1 are depicted as a merge of the brightfield (1) and the AZN-D1 (7) channels.

Figure S4 Cells used for Figure 1A were analyzed by confocal laser-scanning microscopy. Sagital, longitudinal, and transversal two-dimensional sections of a three-dimensional reconstruction are shown. Representative of 10 cells.

Figure S5 Immature monocyte-derived DCs were pre-treated with chloroquine (50–25 µM) for 30 min at 37° and pulsed with AF-488 labeled AZN-D1 (10 µg/ml) for 30 min at 4°C. Next, they were washed and transferred to 37°C for 30 min followed by fixation. Degradation of the ligand was analyzed by flow cytometry, *N* = 3. Data are represented in mean ± SD, a two-way ANOVA was performed.

Figure S6 Representative flow cytometry dot plots of CD8^+^ T cell degranulation.

Figure S7 Immature and lipopolysaccharide-stimulated (*t* = 0) monocyte-derived DCs were treated with the inhibitors: chloroquine (25 µM), MG132 (10 µM), epoxomicin (0.25 µM), cathepsin S inhibitor (5 µM), and 0.1% DMSO (vehicle) for 4 h and thereafter stained with a viability dye and analyzed by flow cytometry. Representative of a *N* = 2.

Figure S8 Immature and lipopolysaccharide-stimulated (*t* = 0) monocyte-derived DCs were incubated with chloroquine (25 µM), MG132 (10 µM), epoxomicin (0.25 µM), cathepsin S inhibitor (5 µM), and 0.1% DMSO (vehicle) for 3 h at 37°C. Thereafter, cells were stained with an α-HLA-A2 antibody and the surface expression of HLA-A2 after inhibitor treatment was analyzed by flow cytometry. Representative of a *N* = 2.

Figure S9 Immature and lipopolysaccharide-stimulated (*t* = 0) monocyte-derived DCs were treated with the inhibitors: chloroquine (25 µM), MG132 (10 µM), epoxomicin (0.25 µM), cathepsin S inhibitor (5 µM), and 0.1% DMSO (vehicle) 30 min prior and during the 3 h antigen pulse with the 9-mer minimal epitope of gp100 peptide. Followed by a co-culture with gp100_280–288_ CD8^+^ T cells for 45 min. The degranulation markers CD107^a/b^ were stained as a measure of CD8^+^ T cell activation, *N* = 3.

Figure S10 Vehicle control of immature and lipopolysaccharide-stimulated (*t* = 0) monocyte-derived DCs. Representative of a *N* = 3.

Figure S11 Immature and lipopolysaccharide-stimulated (*t* = 0) monocyte-derived DCs (moDCs) were incubated 30 min prior and during the 3 h antigen (gp100/AZN-D1) pulse with chloroquine (25 µM), MG132 (10 µM), epoxomicin (0.25 µM), cathepsin S inhibitor (5 µM), and 0.1% DMSO (vehicle). Thereafter, moDCs were co-cultured with gp100_280–288_ CD8^+^ T cells o/n, IFNγ secretion was analyzed by ELISA as a measure for T cell activation. Statistical analysis was performed by executing a one-way ANOVA. Groups are significantly different compared to AZN-D1.
