# Supplementary material for: Toll-Like Receptor 4 Triggering Promotes Cytosolic Routing of DC-SIGN-Targeted Antigens for Presentation on MHC Class I
Source: Front Immunol. 2018 Jun 14;9:1231. doi: 10.3389/fimmu.2018.01231 (PMC6010527; doi:10.3389/fimmu.2018.01231)

Supplementary figures:

S1

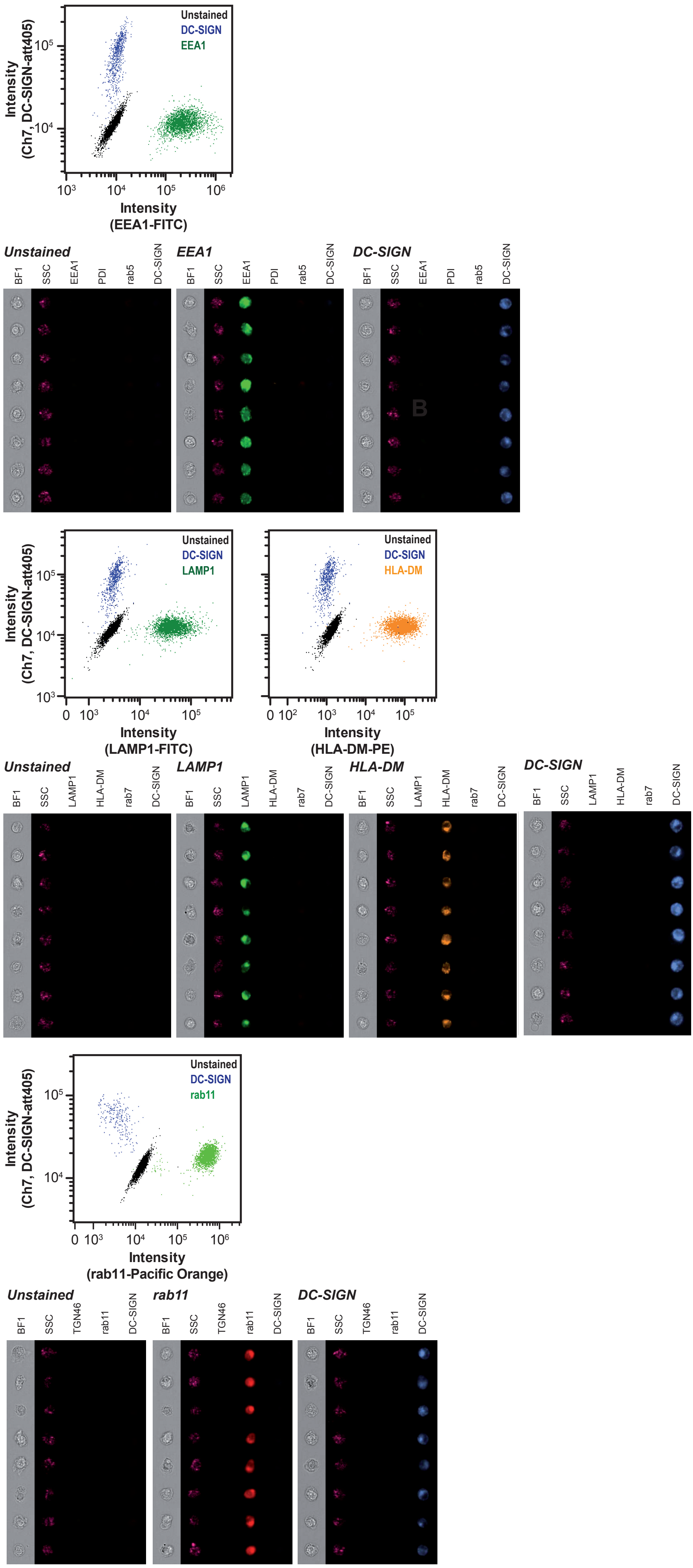

S2

A

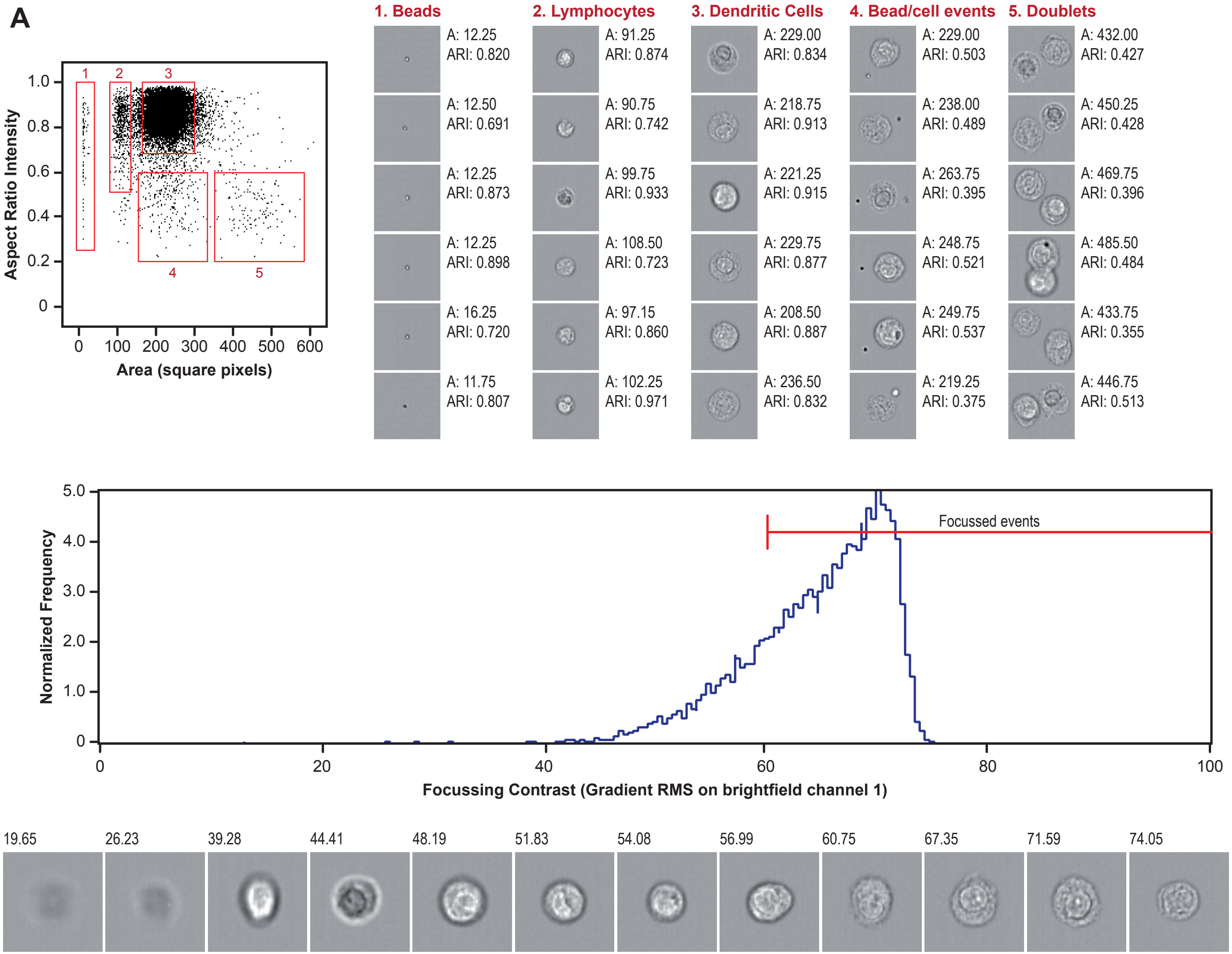

1. Beads
2. Lymphocytes
3. Dendritic Cells
4. Bead/cell events
5. Doublets
Aspect Ratio Intensity
Area (square pixels)
A: 12.25 ARI: 0.820
A: 12.50 ARI: 0.691
A: 12.25 ARI: 0.873
A: 12.25 ARI: 0.898
A: 16.25 ARI: 0.720
A: 11.75 ARI: 0.807
A: 91.25 ARI: 0.874
A: 90.75 ARI: 0.742
A: 99.75 ARI: 0.933
A: 108.50 ARI: 0.723
A: 97.15 ARI: 0.860
A: 102.25 ARI: 0.971
A: 229.00 ARI: 0.834
A: 218.75 ARI: 0.913
A: 221.25 ARI: 0.915
A: 229.75 ARI: 0.877
A: 208.50 ARI: 0.887
A: 236.50 ARI: 0.832
A: 229.00 ARI: 0.503
A: 238.00 ARI: 0.489
A: 263.75 ARI: 0.395
A: 248.75 ARI: 0.521
A: 249.75 ARI: 0.537
A: 219.25 ARI: 0.375
A: 432.00 ARI: 0.427
A: 450.25 ARI: 0.428
A: 469.75 ARI: 0.396
A: 485.50 ARI: 0.484
A: 433.75 ARI: 0.355
A: 446.75 ARI: 0.513
Normalized Frequency
Focussed events
Focussing Contrast (Gradient RMS on brightfield channel 1)
19.65
26.23
39.28
44.41
48.19
51.83
54.08
56.99
60.75
67.35
71.59
74.05

S3

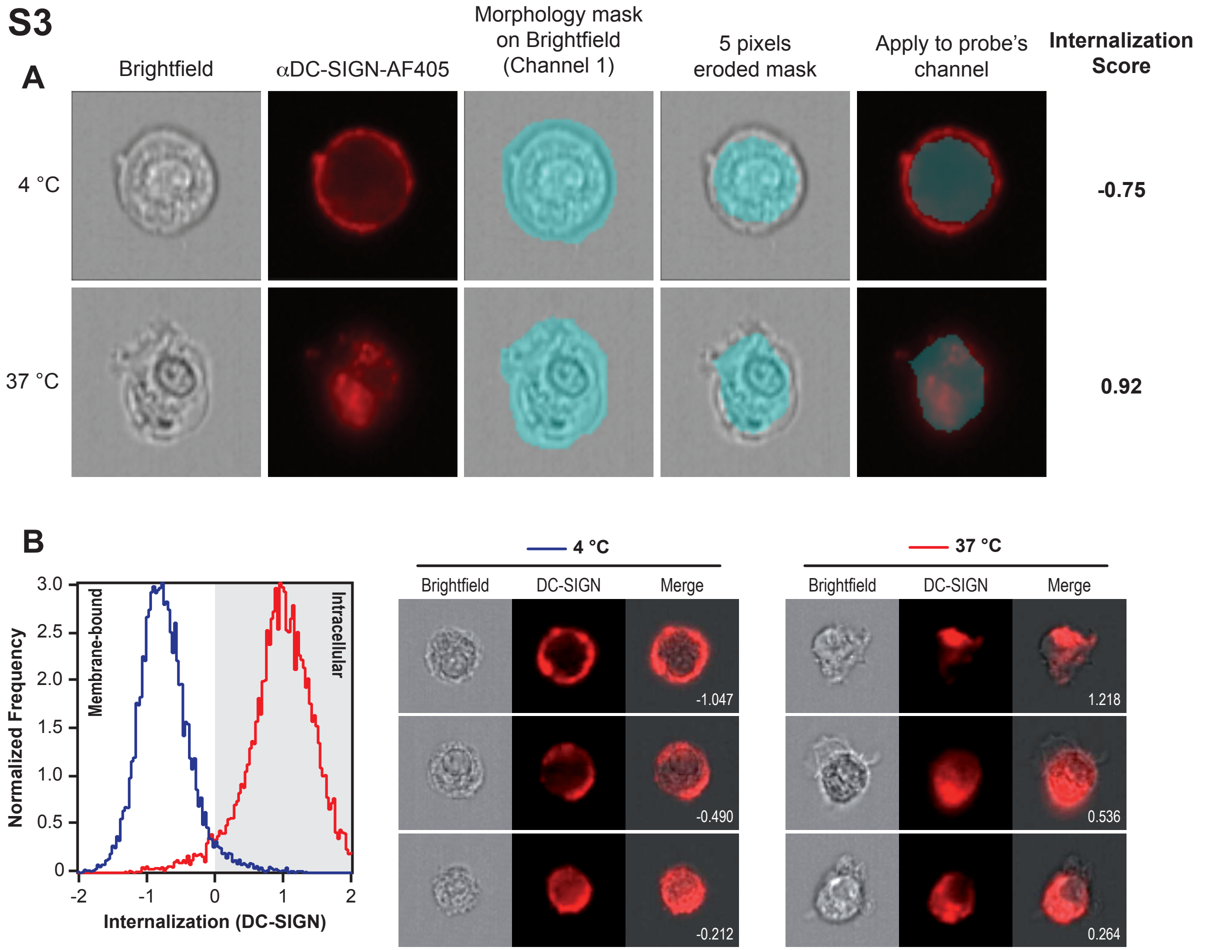
A
Brightfield
αDC-SIGN-AF405
Morphology mask on Brightfield (Channel 1)
5 pixels eroded mask
Apply to probe's channel
Internalization Score
4 °C
-0.75
37 °C
0.92
B
Normalized Frequency
3.0
2.5
2.0
1.5
1.0
0.5
0
Membrane-bound
Intracellular
-2
-1
0
1
2
Internalization (DC-SIGN)
4 °C
37 °C
Brightfield
DC-SIGN
Merge
-1.047
-0.490
-0.212
1.218
0.536
0.264

S4

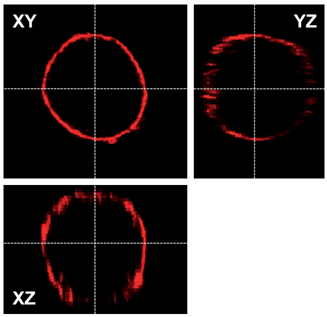
XY
YZ
XZ

S5

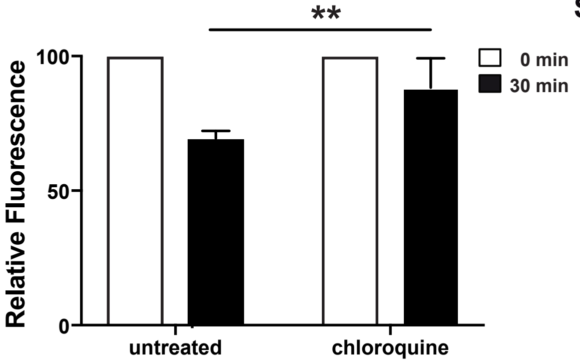

S6

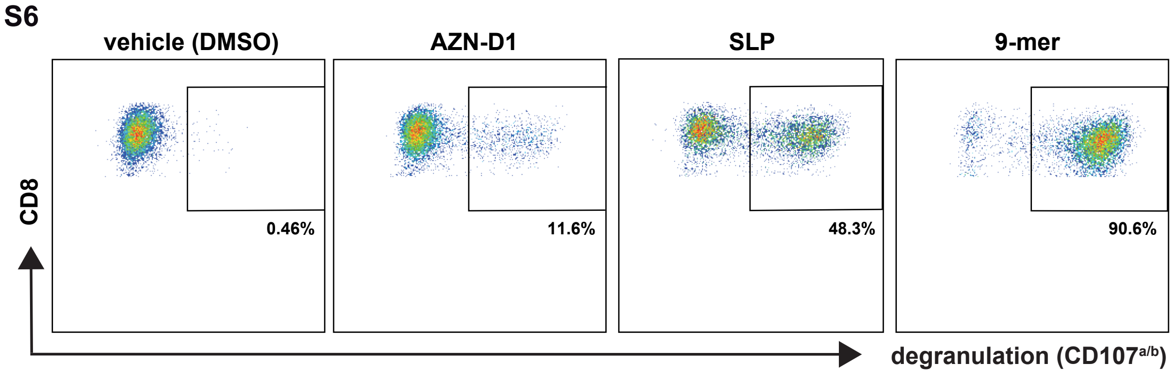

S7

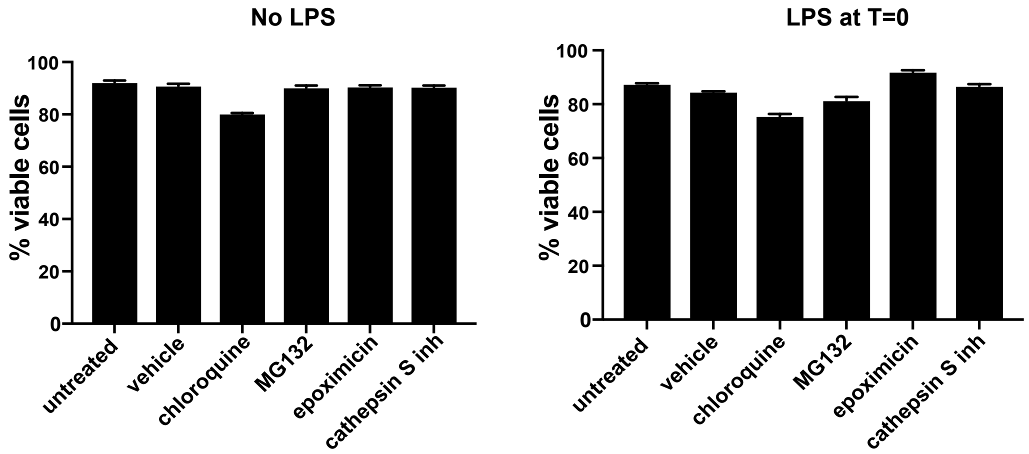

S8

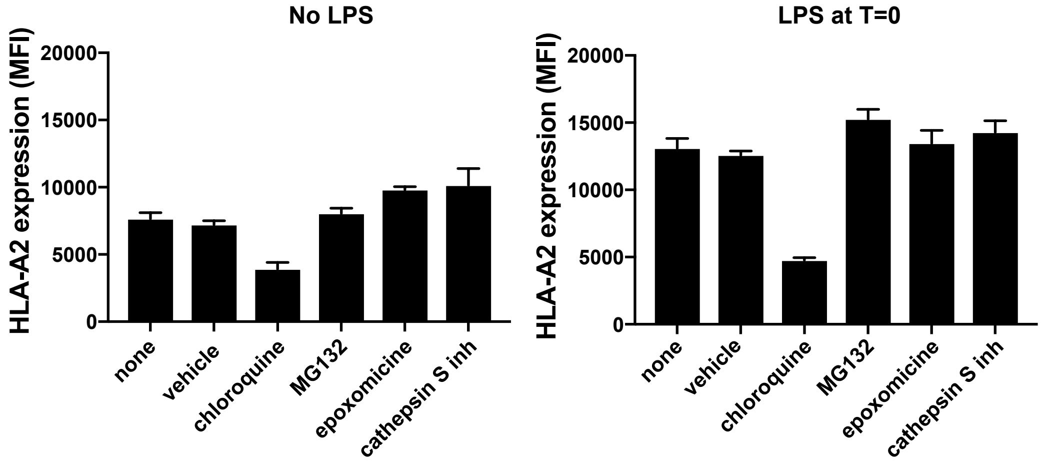

S9

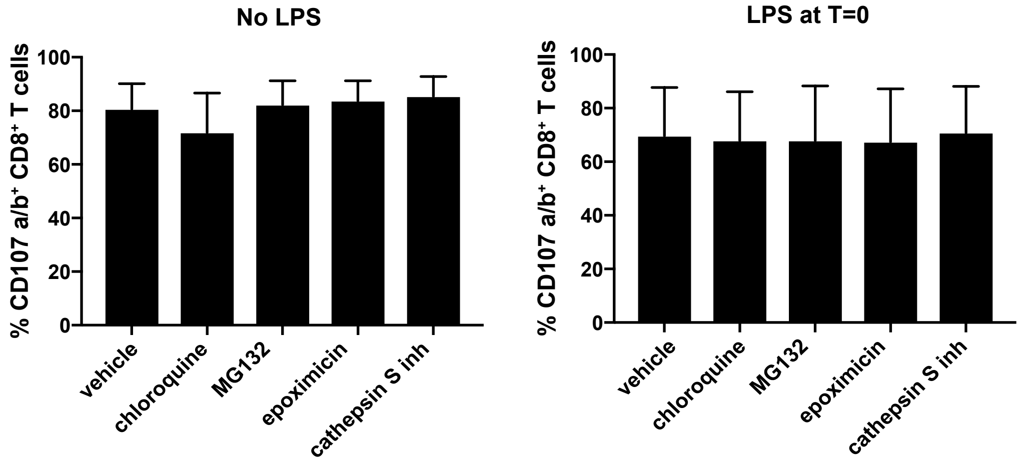

S10

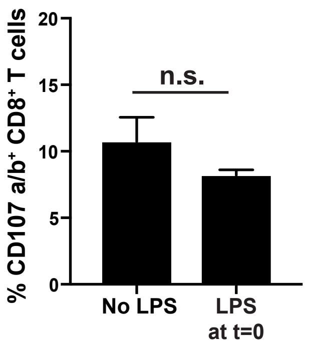

S11

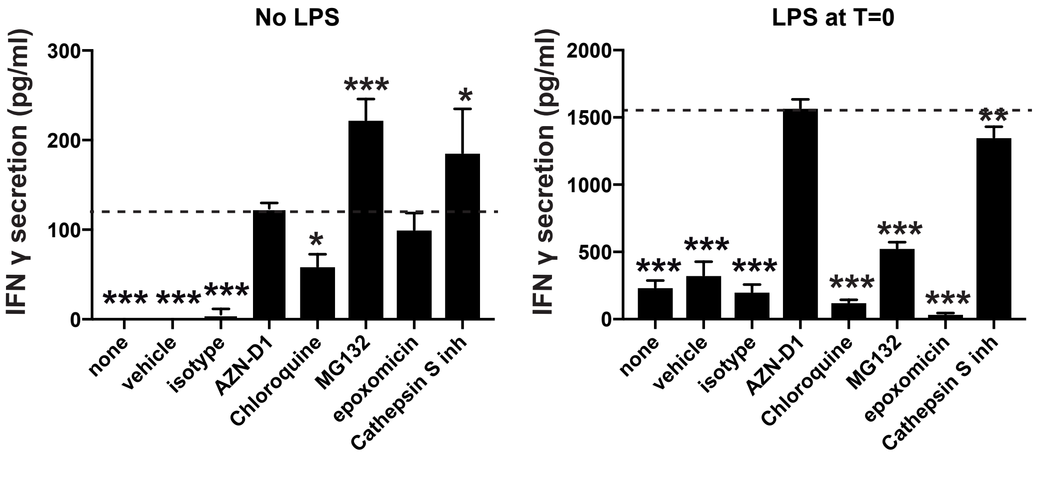

Supplement: Figure S1 — Monocyte-derived DCs (moDCs) were fixed and measured by imaging flow cytometry. Once the compensation table was calculated for each of the staining sets, it was applied to the single staining samples that were acquired using the same settings as experimental samples. Proper compensation was then verified by visualizing samples in bivariate fluorescence intensity plots. Representative images are displayed underneath the corresponding dotplots. [file presentation_1.PDF]
